# Asymmetric Encoder-Decoder Structured FCN Based LiDAR to Color Image Generation

**DOI:** 10.3390/s19214818

**Published:** 2019-11-05

**Authors:** Hyun-Koo Kim, Kook-Yeol Yoo, Ju H. Park, Ho-Youl Jung

**Affiliations:** 1 Department of Information and Communication Engineering, Yeungnam University, Gyeongsan 38544, Korea; kim-hk@ynu.ac.kr (H.-K.K.); kyoo@ynu.ac.kr (K.-Y.Y.); 2 Department of Electrical Engineering, Yeungnam University, Gyeongsan 38544, Korea; jessie@ynu.ac.kr

**Keywords:** advanced driver assistance system, asymmetric network model, image generation, LiDAR sensor, LiDAR imaging

## Abstract

In this paper, we propose a method of generating a color image from light detection and ranging (LiDAR) 3D reflection intensity. The proposed method is composed of two steps: projection of LiDAR 3D reflection intensity into 2D intensity, and color image generation from the projected intensity by using a fully convolutional network (FCN). The color image should be generated from a very sparse projected intensity image. For this reason, the FCN is designed to have an asymmetric network structure, i.e., the layer depth of the decoder in the FCN is deeper than that of the encoder. The well-known KITTI dataset for various scenarios is used for the proposed FCN training and performance evaluation. Performance of the asymmetric network structures are empirically analyzed for various depth combinations for the encoder and decoder. Through simulations, it is shown that the proposed method generates fairly good visual quality of images while maintaining almost the same color as the ground truth image. Moreover, the proposed FCN has much higher performance than conventional interpolation methods and generative adversarial network based Pix2Pix. One interesting result is that the proposed FCN produces shadow-free and daylight color images. This result is caused by the fact that the LiDAR sensor data is produced by the light reflection and is, therefore, not affected by sunlight and shadow.

## 1. Introduction

Light detection and ranging (LiDAR) sensors are widely used for measuring the distances to objects and their reflection information. The distance is calculated by using the round-trip time (RTT) of the light pulses emitted from the sensors. The sensors also provide the reflection intensity, which depends on the materials of the objects. The data obtained from the sensors are the locations of objects in 3D space and their reflection intensities, so the data are often called LiDAR 3D point-cloud data.

Because the LiDAR 3D point-cloud data, namely the range (or distance) and reflection, is independent of sunlight and shadows, the same data can be obtained whether it is day or night [1,2,3,4,5,6,7]. This environmental consistency of LiDAR data has a great advantage over conventional camera images for autonomous vehicle application because the quality of camera images is highly dependent on illumination [8].

One serious problem is that the LiDAR data is too sparse directly to use for the color image generation. To compensate for the sparsity of the LiDAR data, various attempts have been made in the literature. By incorporating the information from camera images, the accuracy of the distance map from LiDAR data is successfully improved in [9,10,11]. However, these fusion methods do not work well at night because the camera image has very low contrast in low-illumination environments. In the same way, the methods produce poor performance if there are many shadows, even during the day. Another approach for solving sparsity is directly to apply the interpolation methods to 2D reflection intensity data [8,12,13]. Because the interpolated image is very noisy, it does not have enough quality for use with conventional computer vision algorithms for object detection and recognition.

Recently, deep-learning-based methods have been applied to various image processing applications such as depth-map generation for stereo matching [14], image reconstruction [15,16], and human action recognition [17]. Moreover, fully convolutional network (FCN) and generative adversarial network (GAN) have provided innovative results on restoring or generating color images from limited information [18,19,20,21,22,23,24,25,26,27]. Particularly, the image is successfully generated from extremely limited information like a sketch [23,25]. Another interesting result is that specific sensor data is generated from different kinds of sensor data [7,26,27].

The innovative results of deep-learning-based methods yield an important insight: the image can be generated from very limited information and/or from different kinds of sensing data. Inspired by these insights, we propose a method that generates color image from the LiDAR 3D reflection data. In our case, the source is a reflection image but the target to be generated is a color image, i.e., different type of data. The proposed method is composed of two steps; (1) the projection of LiDAR 3D reflection data into a 2D reflection image by using geometric configuration and camera parameters, and (2) image generation network to generate the color image from the projected reflection image. For the second step, an FCN-based image-generation network model with an encoder-decoder structure is applied because the model efficiently generates images with low complexity. In our case, the projected reflection image is very sparse and different kind of data with target image. To provide better expression ability at the decoder side, an asymmetrically structured FCN, i.e., the number of layers at the decoder is larger than that at the encoder, is proposed. Notice that the conventional FCN-based image colorization network has normally symmetric structure [19,20,21,22,23,24]. The performance of the proposed LiDAR-to-color image-generation method is evaluated and analyzed by using the peak signal-to-noise ratio (PSNR) [28] and structural similarity index measure (SSIM) [28,29]. In addition, the proposed method is compared with conventional interpolation methods of LiDAR 3D reflection intensity. One interesting simulation result is that the proposed method produces shadow-free and daylight color images under heavily shadowed environments.

The rest of this paper is organized as follows. Section 2 discusses the previous related research. In Section 3, we describe the proposed method to generate a 2D RGB color image from the LiDAR 3D reflection intensity. In Section 4, the proposed asymmetric network structures are empirically analyzed for various depth combinations for the encoder and decoder in the FCN. The performance of the proposed method is compared with the conventional interpolation methods and GAN based Pix2Pix. Section 5 draws the conclusions.

## 2. Related Works

In this section, we describe previous methods related to research on conventional interpolation of LiDAR 3D reflection intensity [8,12,13] and deep-learning-based color image generation [18,19,20,21,22,23,24,25,26,27].

### 2.1. Conventional Interpolation Methods

Ashraf et al. [8] proposed an adaptive interpolation method for LiDAR range data. A 2D interpolated reflection-intensity image is obtained from the LiDAR 3D reflection intensity using various interpolation methods, such as natural neighbor, nearest neighbor, bilinear, bi-cubic, inverse distance weighted, and kriging, and is compared with the corresponding camera-captured gray image to select the best interpolation method. The best method is applied for the range data interpolation. They empirically showed that the inverse distance-weighted interpolation method has the best performance. Chen et al. [12] applied the 2D interpolated reflection intensity image with a camera-based RGB image for lane detection. Asvadi et al. [13] also applied the interpolated reflection-intensity image with a color image for vehicle detection and compared the natural neighbor, nearest neighbor, and bilinear interpolation methods. The nearest neighbor interpolation method has the best performance for vehicle detection.

Even though various existing interpolation methods have been applied to reconstruct the reflection-intensity image, interpolated images still have very poor quality and are affected by severe noise. In addition, color image generation from the reflection intensity has not been reported in the literature.

### 2.2. Color Image-Generation Methods

Since the first fully automatic image colorization method was implemented by using a simple neural network [18], various studies on color image generation using end-to-end deep-learning networks have been reported [19,20,21,22,23,24,25,26,27]. The deep-learning-based color image generation methods commonly use an encoder-decoder-structured FCN [30,31]. The encoder network consists of a combination of a convolution layer, batch-normalization layer [32], dropout [33], activation function, and sub-sampling. The decoder network consists of a combination of up-sampling, convolution layer, batch-normalization layer, dropout, and activation function. The color image-generation network has been applied to a variety of applications, such as converting gray images to color images [19,20,21,22,23,24], sketch images to color images [23,25], and infrared images to color images [26,27].

**Figure 1:**
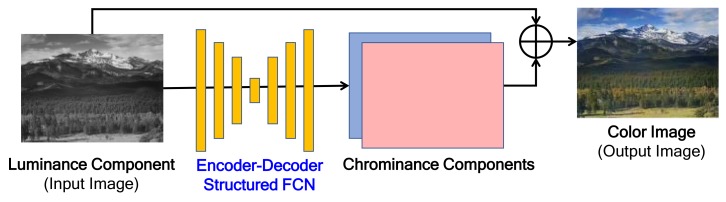
Typical network architecture for gray-to-color image generation.

Figure 1 shows the typical network architecture for gray-to-color image generation [19,20]. The 2-channel chrominance components are predicted from the input single channel luminance component by using the encoder-decoder-structured FCN. Finally, a color image is generated by combining the predicted chrominance components with the input luminance [19,20,21]. Most encoder-decoder-structured FCN have the same number of encoder layers and decoder layers, i.e., symmetric structure.

## 3. Proposed Color Image-Generation System

**Figure 2:**
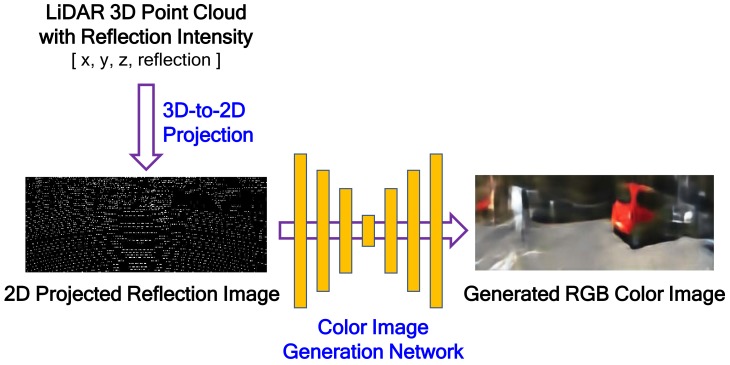
The proposed color image-generation system architecture.

The proposed color image-generation system from LiDAR reflection intensity is composed of two steps: 3D-to-2D projection and color image generation, as shown in Figure 2. The 3D-to-2D projection reconstructs a 2D projected reflection image by projecting the LiDAR 3D reflection intensity onto the target image plane desired. The projected reflection image is very sparse, as shown in lower left picture of Figure 2, because of the different resolution and field of view (FOV) between LiDAR and the target image. The target image is assumed to have the same image plane that is captured by a camera installed on the vehicle. The goal of our work is to generate a color image that is as similar as possible to the image captured by the camera from the LiDAR 3D reflection intensity. In the image-generation step, RGB components are generated from the 2D reflection image using the encoder-decoder-structured FCN model. Unlike the conventional FCN application, the color image should be generated from the sparse 2D reflection image. This is why the proposed FCN is designed to have an asymmetric network structure, i.e., the layer depth of the decoder in the FCN is deeper than that of the encoder.

In the following subsection, we describe in detail the 3D-to-2D projection and color image- generation network. In addition, the training and inference processes are also described.

### 3.1. 3D-to-2D Projection

The 2D reflection intensity image is reconstructed from the LiDAR 3D point cloud by using a LiDAR-to-camera projection matrix, which maps the reflection intensity value of LiDAR data onto the corresponding image coordinates [8,13,34].

### 3.2. Proposed Color Image-Generation Network

**Figure 3:**
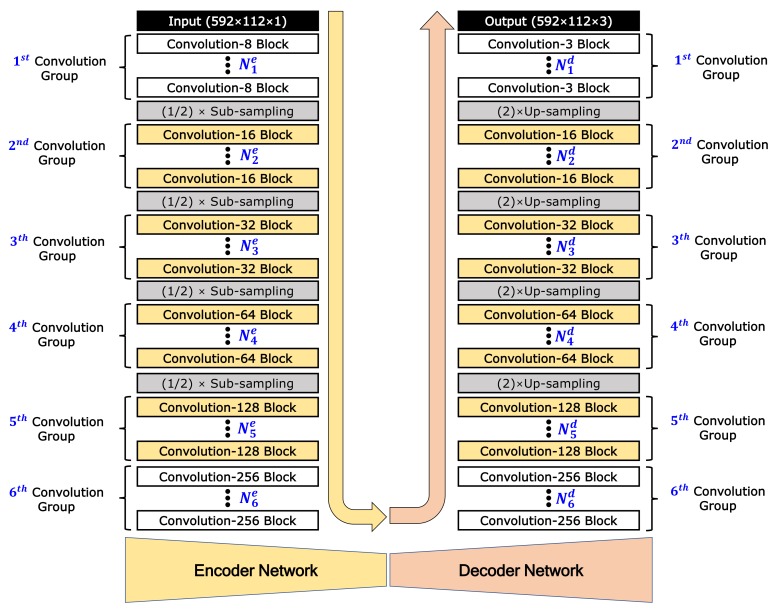
Architecture design of our proposed image-generation network model.

The proposed color image-generation network model is designed with an asymmetric encoder-decoder-structured FCN model as shown in Figure 3. This model generates a color image with 3 channels (size: 592 × 112 × 3) from the sparse 2D reflection image with 1 channel (592 × 112 × 1). The encoder network consists mainly of several convolution blocks and sub-sampling steps. Each convolution block is composed of a convolution layer, batch-normalization layer, and activation function, in consecutive order. The decoder also consists of several up-sampling steps and convolution blocks. As various numbers of convolution blocks can be applied before each sub-sampling or up-sampling, we introduce a new terminology called the convolution group, which consists of several convolution blocks. In this work, considering the size of the input and output images, the encoder network is designed with six convolution groups and four sub-sampling steps. The decoder is designed with four up-sampling steps and six convolution groups. In Figure 3, $N_{i}^{e}$ and $N_{i}^{d}$ represent the number of convolution blocks in the convolution group of the encoder and decoder, respectively. As results, the number of convolution blocks is $\sum_{i=1}^{6}N_{i}^{e}$ and $\sum_{i=1}^{6}N_{i}^{d}$ in the encoder and decoder networks, respectively. For sub-sampling, max-pooling with a factor of 2 is applied. For up-sampling, un-pooling [35] with a factor of 2 is applied. In each block, *K* convolution layers are applied, which is denoted as the convolution-*K* block. According to the research results that indicate that dropout is not needed when using batch normalization [32], dropout is not applied in the proposed network.

Whereas the convolution-8 block is applied in the first convolution group of the encoder, the convolution-3 block is applied in the last convolution group of the decoder because the output is a 3-channel color image. In all the convolution layers, stride 1 and the same zero padding are applied. Except for the convolution-3 blocks of the last convolution group in the decoder, a rectified linear unit (ReLU) is used for the activation function in all convolution blocks. In the last convolution-3 blocks, hyperbolic tangent (tanh) is used for the activation function and batch normalization is not applied. The ReLU and tanh functions are as follows:

(1) ReLU(x)=max(0,x),

(2) $$\tanh\left(x\right)=2\left(\frac{1}{1+e^{-2x}}\right)-1.$$

**Figure 4:**
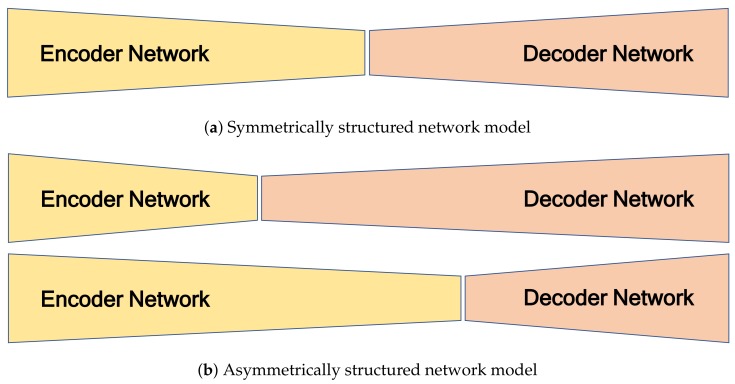
Encoder-Decoder-structured FCN models.

The role of the encoder network is to extract features from the sparse 2D reflection-intensity image. The role of the decoder network is to map the low-resolution feature maps to an RGB color image with full output resolution. Because the projected 2D reflection and generated color images have different amounts of information, it is necessary to apply different numbers of convolution blocks in the encoder and decoder. The proposed network can be regarded as a symmetrically structured FCN when it has the same the number of blocks between the *i*th convolution groups in the encoder and decoder ($N_{i}^{e}=N_{i}^{d}$) as shown in Figure 4a. An asymmetrically structured FCN can be realized if the *i*th convolution groups in the encoder and decoder have different numbers of block ($N_{i}^{e}\neq N_{i}^{d}$). Figure 4b shows two cases of asymmetrically structured networks: the first is the case of a decoder that has greater depth than the encoder ($\sum_{i=1}^{6}N_{i}^{e}>\sum_{i=1}^{6}N_{i}^{d}$), and the second is the reverse ($\sum_{i=1}^{6}N_{i}^{e}<\sum_{i=1}^{6}N_{i}^{d}$).

The total number of layers in the proposed network is obtained by summing the number of convolutions and batch-normalization layers in both the encoder and decoder. As each convolution block consists of one convolution and one batch-normalization layer, the number of layers at encoder $L^{e}$ and decoder $L^{d}$ are calculated as follows:

(3) $$L^{e}=2\sum_{i=1}^{6}N_{i}^{e},$$

(4) $$L^{d}=2\sum_{i=1}^{6}N_{i}^{d}-N_{6}^{d}.$$

Note that batch normalization is not performed in the last convolution block of the decoder. Therefore, the number of total layers $L^{t}$ is calculated as follows:

(5) $$\begin{array}{cc} L^{t} & =L^{e}+L^{d}\\ \; & =2\sum_{i=1}^{6}\left(N_{i}^{e}+N_{i}^{d}\right)-N_{6}^{d}. \end{array}$$

Assume that all convolution filters used in the proposed network have the same size (F×F). As all convolution blocks in the *i*th convolution group have the same number of convolution filters, each block belonging to the *i*th group has $K_{i}^{e}$ (or $K_{i}^{d}$) convolution filters in the *i*th group of the encoder (or decoder). The total number of parameters is obtained by summing the number of weights and biases of the convolution layers and the number of parameters of the batch-normalization layers. The number of parameters at encoder $M^{e}$ is calculated as follows:

(6) $$M^{e}=F^{2}\left[K_{1}^{e}+\sum_{i=1}^{6}{\left(K_{i}^{e}\right)}^{2}\left(N_{i}^{e}-1\right)+\sum_{i=1}^{5}K_{i}^{e}K_{i+1}^{e}\right]+\sum_{i=1}^{6}K_{i}^{e}N_{i}^{e}+4\sum_{i=1}^{6}K_{i}^{e}N_{i}^{e}.$$

At the decoder, the number of parameters $M^{d}$ is given by:

(7) $$M^{d}=F^{2}\left[K_{6}^{e}K_{6}^{d}+\sum_{i=1}^{6}{\left(K_{i}^{d}\right)}^{2}\left(N_{i}^{d}-1\right)+\sum_{i=1}^{5}K_{i}^{d}K_{i+1}^{d}\right]+\sum_{i=1}^{6}K_{i}^{d}N_{i}^{d}+4\sum_{i=2}^{6}K_{i}^{d}N_{i}^{d}.$$

In Equations (6) and (7), the first and second terms on the right side indicate the number of weights and biases in the convolution layers and the third term is the number of parameters in the batch-normalization layers. Therefore, the total number of parameters in the proposed network $M^{t}$ is given by:

(8) $$M^{t}=M^{e}+M^{d}.$$

As mentioned before, the number of convolution filters in the first group of the encoder and the last group of the decoder are fixed to eight and three, i.e., $K_{1}^{e}=8$ and $K_{1}^{d}=3$. For other groups, we design that the number of convolution filters is increased by a power of two ($K_{i}^{e}=K_{i}^{d}=2^{i+2}$, for 2≤i≤6). All convolution filters are designed to be of (3 × 3) size (F=3).

### 3.3. Training and Inference Processes

**Figure 5:**
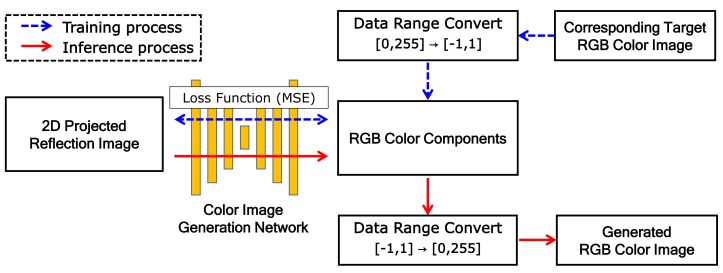
Training and inference processes in the proposed network.

Figure 5 shows the training and inference processes of the proposed network. The training process is indicated by blue dashed arrows and the inference process by red solid arrows.

In the training process, the 2D projected reflection-intensity image and corresponding RGB color image are used as the dataset. The projected reflection image is used as input for the proposed model and the corresponding color image is used as the target image that is the ground truth (GT). Because the tanh function is used as the activation function of the last convolution group, the dynamic range of the output image to be generated is [−1,1]. Thus, the GT color image is converted to the same dynamic range, where each color component is mapped to the dynamic range independently. The loss function is mean-squared error (MSE) between target *T* and generation *G* images, as follows:

(9) $$MSE=\frac{1}{mnc}\sum_{i=0}^{m-1}\sum_{j=0}^{n-1}\sum_{k=0}^{c-1}{\left[T\left(i,j,c\right)-G\left(i,j,c\right)\right]}^{2},$$

where, *m* and *n* are the width and height of the image and *c* indicates the number of channels.

In the inference process, RGB components are generated through the proposed color image-generation network with training parameters. As the RGB components have a dynamic range of [−1,1], the final generated RGB color image is obtained by conversion to the range of [0,255].

## 4. Experimental Results

In this section, we describe the configuration of the dataset for the simulation, the hyper- parameters for learning, and the quality measure metrics. We evaluate the performance of the proposed color image generation with varying depths of the encoder and decoder networks. The proposed method is also compared with conventional interpolation methods and GAN based Pix2Pix [23].

### 4.1. Evaluation Dataset

For simulations, our dataset is reconstituted from the raw KITTI dataset [34], which is recorded under various driving environments such as road, city, and residential areas during the day. For time consistency, we combine a pair of LiDAR 3D reflection intensity and stereo color image that are captured at the same time. A Velodyne HDL-64E rotating 3D laser scanner is used [4]. The 2D projected reflection image is obtained by projecting the LiDAR 3D reflection intensity onto the target image plane as mentioned in Section 3.1. The corresponding color image is obtained by randomly selecting one of the left and right images. Paired images with the 2D projected reflection and color image are used as the evaluation dataset for the color image-generation networks. When constructing the evaluation datasets, we manually exclude image pairs recorded under heavy shadows in order to generate shadow-free color images. Both the 2D projected reflection and color images are cropped to size of 1184 × 224 so that they have the same valid area. These are then resized into 592 × 112 by sub-sampling for simplicity of simulation. The evaluation dataset consists of 4308 image pairs, which are 2872 pairs for training, 718 for validation, and 718 for testing. The three sets have similar distributions of scene categories (city, residential, and road), color camera positions (left and right), and temporal index. Detailed descriptions of the evaluation dataset are summarized in Table 1. The number of image pairs for training, validation, and testing are listed and the numbers of left and right images are indicated separately.

**Figure 6:**
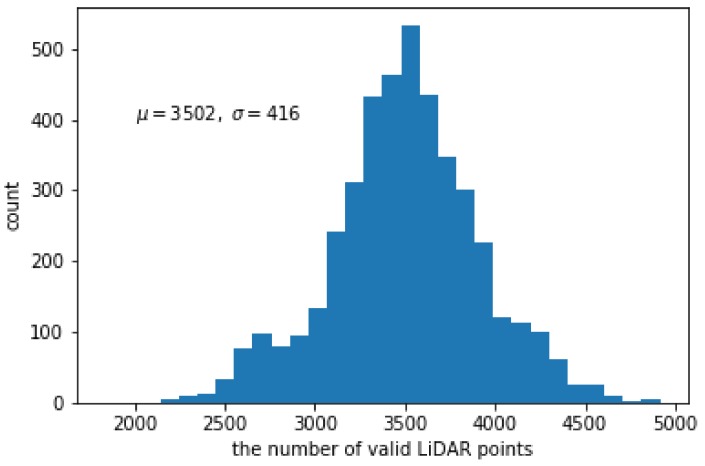
Histogram of the number of valid LiDAR points in 2D lidar reflection-intensity image.

Figure 6 shows the histogram of the number of valid LiDAR points in the 2D projected reflection image. The maximum and minimum numbers of valid points are 4916 (7.41%) and 1832 (2.76%). In average, the number of points is 3502, which means the sparseness ratio is 5.28%. The reflection images are very sparse and even irregular compared to the target color image.

### 4.2. Hyper-Parameters for Training

Because the amount of information on the 2D projected reflection image is very small, the number of blocks in the first convolution group of the proposed image-generation network is fixed to one ($N_{1}^{e}=1$). Accordingly, the final group has also one convolution block ($N_{1}^{d}=1$). The last encoder group and the first decoder group are also set to have one convolution block ($N_{6}^{e}=N_{6}^{d}=1$). The remaining convolution groups are designed with different numbers of convolution blocks. For simplicity of the performance evaluation, however, we assume that these groups have the same number of blocks in the encoder and decoder, respectively, that is $N_{i}^{e}≜N^{e}$ and $N_{i}^{d}≜N^{d}$ for i=2,3,4,5. The performance of the proposed network are evaluated with different values of $N^{e}$ and $N^{d}$. Hereinafter, we use the notation M-$N^{e}$-$N^{d}$, which denotes the proposed network model with block numbers $N^{e}$ and $N^{d}$.

The proposed network is trained until the maximum of 2000 epochs. During training, an adaptive moment estimation solver, called Adam [36], is applied with batch size 4, learning rate $5\times 10^{-4}$, and momentum parameters $\beta_{1}$ = 0.9, $\beta_{2}$ = 0.999, and ϵ = $10^{-8}$.

### 4.3. Measurement Metrics

To evaluate the performance of the generated image, PSNR [28] and SSIM [28,29] are used. The PSNR between the generated color and GT images is calculated based on the RGB components as follows:

(10) $$PSNR=10\log_{10}\left(\frac{255^{2}}{MSE}\right),$$

where MSE is given in Equation (9).

SSIM is calculated using only the gray-scale *Y* component [37], as follows:

(11) $$SSIM=\frac{\left(2\mu_{G}\mu_{T}+C_{1}\right)\left(2\sigma_{GT}+C_{2}\right)}{\left(\mu_{G}^{2}+\mu_{T}^{2}+C_{1}\right)\left(\sigma_{G}^{2}+\sigma_{T}^{2}+C_{2}\right)},$$

where $\mu_{G}$ and $\mu_{T}$ represent the average of the generated and target images, $\sigma_{G}^{2}$ and $\sigma_{T}^{2}$ are variances, and $\sigma_{GT}$ is covariance. The positive constants $C_{1}\left(=0.0001\right)$ and $C_{2}\left(=0.0009\right)$ are used to avoid a null denominator.

### 4.4. Experiments with Symmetrically Structured Network

In this subsection, we analyze the performance of symmetrically structured networks, which consist of the same number of convolution blocks in both the encoder and decoder ($N^{e}=N^{d}$). When varying the number of blocks from one to ten, the numbers of layers and of parameters are calculated as listed in Table 2. Note that M-$N^{e}$-$N^{d}$ denotes a network with $N^{e}$ and $N^{d}$ blocks at each convolution group (i=2,3,4,5). The encoder has one more layer than the decoder because the sixth group of the decoder does not have a batch-normalization layer. The decoder has more parameters than the encoder because of the increasing filter dimension.

Table 3 shows the performance results according to the total number of layers in the symmetric model. For the case of the symmetric structure, the proposed network with 103 layers, i.e., M-6-6 ($N^{e}$ = $N^{d}$ = 6), produces the best performance on average in terms of both PSNR and SSIM. If the network has more than 103 layers, the vanishing-gradient-problem [38] occurs, resulting in poor performance.

### 4.5. Experiments with Asymmetrically Structured Network

In this subsection, we analyze asymmetrically structured networks, which consist of different numbers of convolution blocks in the encoder and decoder. First, the numbers of layers and parameters are examined and listed in Table 4. This shows that the number of parameters as well as the number of layers are not changed when the sum of $N^{e}$ and $N^{d}$ is constant. For example, M-3-9, M-6-6, and M-9-3 have the same number of total layers (103) and the number of total parameters (3,350,243).

**Figure 7:**
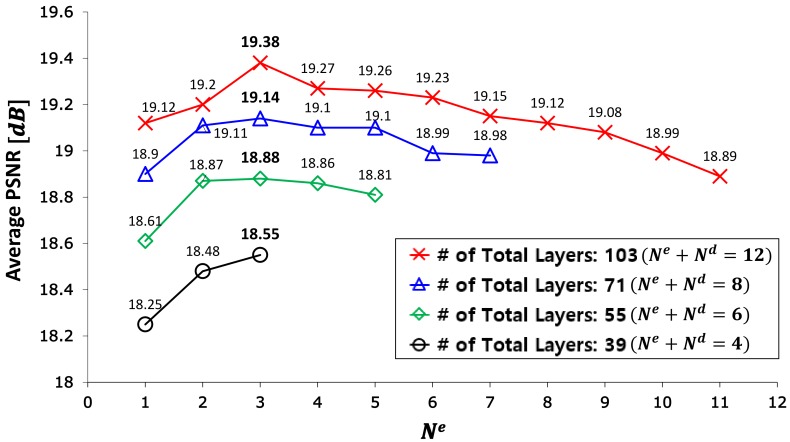
PSNR performance according to $N^{e}$ in a model with a fixed total number of layers.

Figure 7 shows the PSNR performance results with respect to $N^{e}$ for the case of using four different total numbers of layers. For example, the red color curve with cross shows the average PSNR over different $N^{e}$ when $N^{e}+N^{d}=12$, i.e., when using a total of 103 layers. M-3-9 ($N^{e}=3$) achieves the best performance. In other cases, $N^{e}=3$ also achieves the best performance. In general, it is not necessary for the coverage of convolution filter to be much larger than the input image. For the encoder network, the filter coverage is determined by the number of sub-sampling $R_{sp}$ and the number of convolution blocks. Considering the encoder, except for the first and the last convolution groups, the coverage of convolution filters can be derived with respect to the input image size when using (3 × 3) filters. It is reasonable that the coverage is not greater than the size of the input image, as follows:

(12) $$2^{\left(R_{sp}\right)}\left(2N^{e}+1\right)\leq \min\left(m,n\right),$$

where *m* and *n* are the width and height of the image. In this equation, the left term is the coverage of the convolution filters.

For our case, the image size is (592 × 112) and the coverage of filters is 112 when $N^{e}=3$. This can be why the performance decreases when $N^{e}$ is greater than 3. From Figure 7, it is observed that the performance can increase when the layer depth of the decoder is deeper than that of the encoder. For example, the red color curve with cross has better performance than the blue color curve with triangle at the same $N^{e}$. Typically, M-3-9 has higher average PSNR than M-3-5. It is necessary to analyze in detail the performance according to the depth of the decoder.

Table 5 shows the performance in terms of PSNR and SSIM according to the depth of the decoder, where the depth of the encoder is fixed to 28 layers ($N^{e}=3$). The performance monotonically increases until $N^{d}$ reaches nine. It decreases when $N^{d}$ is greater than nine. In particular, it drops abruptly from $N^{d}=12$. M-3-9 achieves the best performance. As in the case of the symmetrically structured network, the asymmetric network also has the vanishing-gradient-problem for more than 103 layers.

Even though M-6-6 and M-3-9 have the same numbers of layers and parameters (103 layers and 3,350,243 parameters), M-3-9 has better performance than M-6-6. This is a typical example of the fact that asymmetric networks have better performance than symmetric networks. Therefore, it is recommended that the proposed FCN be designed to have a deeper decoder than encoder for sparse input data such as LiDAR 2D reflection intensity.

### 4.6. Comparison to State-of-the-Art

The two conventional interpolation methods, such as inverse distance weighted (IDW) and nearest neighbor (NN), and GAN based Pix2Pix [23] are tested for the performance evaluation of the proposed method. Because the interpolation results are only gray-scale images, PSNR is calculated using only the gray-scale *Y* component of the GT image. In case of the GAN based Pix2Pix, generator network depth is changed to adapt the spatial resolutions of input and output images used in the simulation. As shown in Table 6, the proposed FCN with M-3-9 has, on average, ’8.23 dB and 3.41 dB’ higher PSNR and ’0.3 and 0.11’ greater SSIM than IDW interpolation and Pix2Pix, respectively.

**Figure 8:**
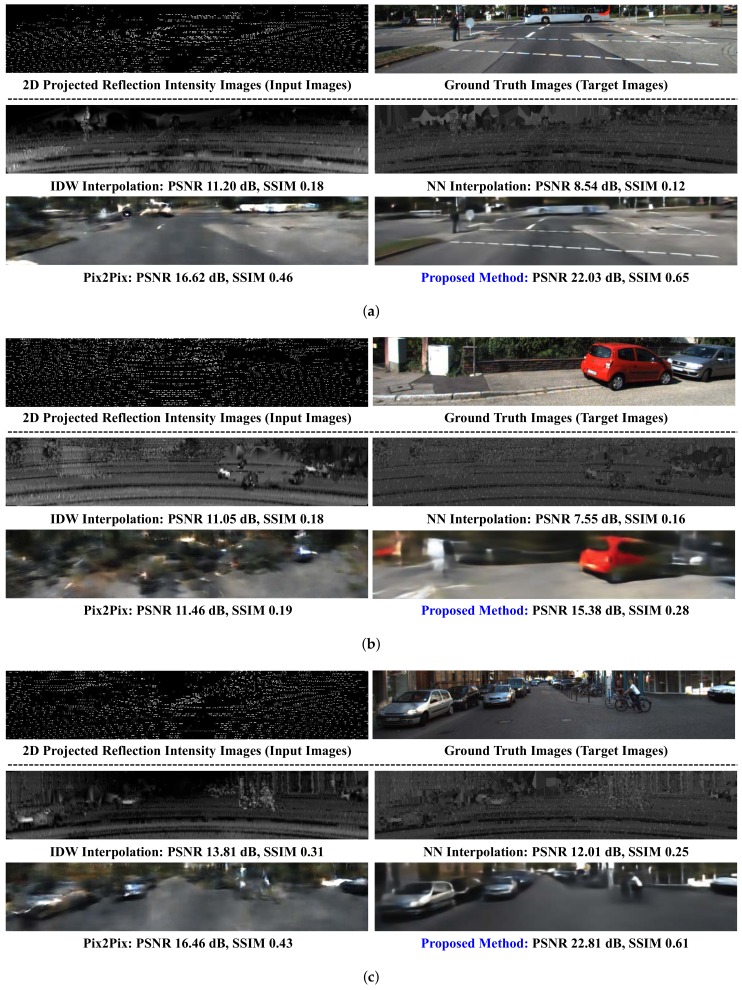
Inference examples in the validation and test dataset. (**a**) Case images with bus and road lanes; (**b**) Case images captured at short distance; and (**c**) Case images with vehicles at various distances are shown.

Figure 8 compares the subjective visual qualities for various methods. In case of Figure 8a, the two interpolation methods cannot generate the object colors in nature. Also, the bus located at the top-middle portion of image is hard to be recognizable. In case of Pix2Pix, objects are generated with their colors but are heavily blurred. One interesting phenomenon in Pix2Pix is that the bus is generated at the different location and that pedestrian is disappeared. On the contrary, the proposed method generates objects at the correct location and with similar colors, compared to others. All the methods produce poor visual qualities with low PSNR and SSIM in Figure 8b. The whole image generated by Pix2Pix is extremely blurred and it is completely unrecognizable. The curbstone is highly blurred and indistinguishable in the proposed method, but other objects are relatively well-generated. Figure 8c shows that IDW generates better objects such as car, bicycle, and person than NN. And the similar trends are observed in others.

**Figure 9:**
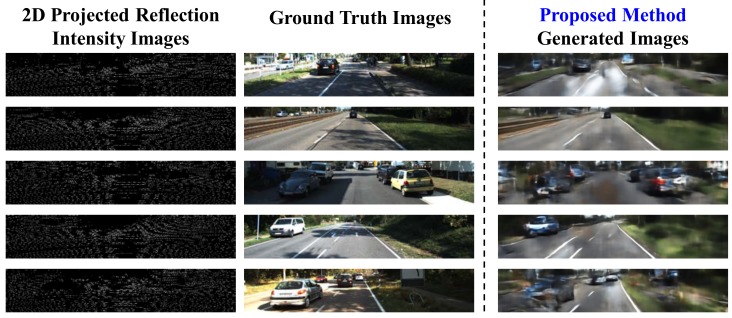
Additional inference examples of GT images with heavy shadows.

Figure 9 shows additional example images generated from LiDAR data that were captured under heavily-shadowed environments. Notice that these heavily-shadowed images are not used in training and validation. Heavy shadows can be seen in the GT images but is almost removed in the generated color images. This means that the proposed FCN-based color image-generation network can generate shadow-free color images from LiDAR sensor data.

## 5. Conclusions

In this paper, we propose a color image-generation method from LiDAR 3D reflection intensity. The proposed method consists of 3D-to-2D projection and an image-generation network. For the image-generation network, an asymmetrically structured FCN is designed considering the sparseness of the projected reflection image.

Through simulations, it is shown that the proposed method generates fairly good visual quality of images while maintaining almost the same color as the GT image. In particular, the asymmetrically structured FCN with a deeper decoder than encoder generates a higher-quality color image. Until the total number of layers reaches a certain number, the quality of the generated image monotonically increases. We also prove that the proposed method produces improvements of ’8.23 dB and 3.41 dB’ in PSNR and ’0.3 and 0.11’ in SSIM over the conventional interpolation methods and Pix2Pix, respectively. In addition, the proposed FCN-based color image-generation network can generate shadow-free color images from LiDAR sensor data. We expect that the proposed method can generate daytime color images at night because the same LiDAR data can be obtained whether it is day or night. This means that the proposed method could be very useful for developing various nighttime driving assistance systems. These results can help developers design FCN-based image-generation networks from very limited information and/or different kinds of sensing data. 

## Figures and Tables

**Table 1:** Evaluation dataset.

Dataset	Category						Total		
	City		Residential		Road				
	Left	Right	Left	Right	Left	Right	Left	Right	Total (Ratio)
Training set	376	374	208	209	852	853	1436	1436	2872 (66.6%)
Validation set	93	95	51	53	213	213	357	361	718 (16.7%)
Testing set	94	93	53	51	214	213	361	357	718 (16.7%)
Total sets	563	562	312	313	1279	1279	2154	2154	4308 (100.0%)

**Table 2:** Number of layers and parameters when varying the number of blocks ($N^{e}$ and $N^{d}$) in symmetric structured networks.

Network Model	Encoder		Decoder		Total	
	$L^{e}$	$M^{e}$	$L^{d}$	$M^{d}$	$L^{t}$	$M^{t}$
M-1-1	12	395,424	11	984,419	23	1,379,843
M-2-2	20	592,464	19	1,181,459	39	1,773,923
M-3-3	28	789,504	27	1,378,499	55	2,168,003
M-4-4	36	986,544	35	1,575,539	71	2,562,083
M-5-5	44	1,183,584	43	1,772,579	87	2,956,163
M-6-6	52	1,380,624	51	1,969,619	103	3,350,243
M-7-7	60	1,577,664	59	2,166,659	119	3,744,323
M-8-8	68	1,774,704	67	2,363,699	135	4,138,403
M-9-9	76	1,971,744	75	2,560,739	151	4,532,483
M-10-10	84	2,168,784	83	2,757,779	167	4,926,563

**Table 3:** Performance results when varying the number of blocks ($N^{e}$ and $N^{d}$) in symmetric structured networks. Bold-faced numbers indicate the top-ranked network model and its scores.

Network Model	Validation Set		Test Set		Total	
	PSNR	SSIM	PSNR	SSIM	PSNR	SSIM
M-1-1	17.45	0.41	17.45	0.41	17.45	0.41
M-2-2	18.50	0.46	18.45	0.46	18.48	0.46
M-3-3	18.90	0.47	18.86	0.48	18.88	0.47
M-4-4	19.12	0.48	19.05	0.48	19.09	0.48
M-5-5	19.20	0.49	19.13	0.49	19.17	0.49
**M-6-6**	**19.23**	**0.49**	**19.22**	**0.49**	**19.23**	**0.49**
M-7-7	19.19	0.48	19.16	0.48	19.18	0.48
M-8-8	19.12	0.48	19.10	0.48	19.11	0.48
M-9-9	18.85	0.47	18.83	0.47	18.84	0.47
M-10-10	18.76	0.46	18.72	0.46	18.74	0.46

**Table 4:** Number of layers and parameters when varying the number of blocks ($N^{e}$ and $N^{d}$) in asymmetric structured networks.

$N^{e}+N^{d}$	$L^{t}$	$M^{t}$
4	39	1,773,923
5	47	1,970,963
6	55	2,168,003
7	63	2,365,043
8	71	2,562,083
9	79	2,759,123
10	87	2,956,163
11	95	3,153,203
12	103	3,350,243
13	111	3,547,283
14	119	3,744,323
15	127	3,941,363
16	135	4,138,403

**Table 5:** Performance according to depth of decoder in the proposed asymmetric structured networks. Bold-faced numbers indicate the top-ranked network model and its scores.

Network Model	Validation Set		Test Set		Total	
	PSNR	SSIM	PSNR	SSIM	PSNR	SSIM
M-3-1	18.55	0.46	18.55	0.46	18.55	0.46
M-3-2	18.69	0.47	18.49	0.47	18.69	0.47
M-3-3	18.90	0.47	18.86	0.47	18.88	0.47
M-3-4	19.02	0.48	19.02	0.48	19.02	0.48
M-3-5	19.15	0.49	19.13	0.49	19.14	0.49
M-3-6	19.17	0.49	19.14	0.49	19.16	0.49
M-3-7	19.27	0.49	19.24	0.49	19.26	0.49
M-3-8	19.36	0.49	19.34	0.50	19.35	0.495
**M-3-9**	**19.40**	**0.50**	**19.36**	**0.50**	**19.38**	**0.50**
M-3-10	19.33	0.49	19.34	0.49	19.34	0.49
M-3-11	19.31	0.49	19.33	0.49	19.32	0.49
M-3-12	18.55	0.45	18.54	0.45	18.55	0.45
M-3-13	18.41	0.44	18.35	0.44	18.38	0.44

**Table 6:** Performance results of the proposed method and conventional methods. Bold-faced numbers indicate the top-ranked method and its scores.

Method	Validation Set		Test Set		Total	
	PSNR	SSIM	PSNR	SSIM	PSNR	SSIM
IDW interpolation	11.15	0.20	11.14	0.20	11.15	0.20
NN interpolation	9.36	0.18	9.35	0.18	9.36	0.18
Pix2Pix	15.96	0.39	15.98	0.39	15.97	0.39
**Proposed method**	**19.40**	**0.50**	**19.36**	**0.50**	**19.38**	**0.50**
